# Observations and Queries on Animal Heat

**Published:** 1786

**Authors:** John Pearson

**Affiliations:** Surgeon to the Lock Hospital, and to the Public Dispensary in Carey Street


### XI. *Obſervations and Queries on Animal Heat. By Mr.* John Pearſon, *Surgeon to the Lock Hoſpital, and to the Public Diſpenſary in Carey Street.*

### SECTION I.

THE phenomena attending heated bodies, and ſubſtances in a ſtate of combuſtion, have attracted the attention of philoſophers in all ages: it was ſoon diſcovered that heat and light are not inſeparable; and a diſtinction was formed between heat and fire, at a very early period, by Theophraſtus in his book *de Igne* *.

The cauſes which operate in the generation of animal heat, and in the preſervation of that nearly equal temperature which the healthy body of a living animal maintains, have long been problems which the moſt ingenious philoſophers and phyſiologiſts have endeavoured to ſolve. The principal grounds upon which they have proceeded may be arranged under three diviſions, viz. that animal heat is to be aſcribed to the efficiency of cauſes, pneumatical, mechanical, or chymical.

Thoſe pneumatical phyſiologiſts who inform us that animal heat is produced by the energy of

* Fire he conſiders as a *principle* or *cauſe*, not that which appeareth to *ſenſe* as a *paſſion* or *accident exiſting in a ſubject.* Siris. page 81.

of the living principle, ſeem to beſtow as much information as thoſe ancients who aſcribed the operation of opium to its *anodyne faculty*, &c. Such modes of explanation are highly inimical to the advancement of ſcience, by encouraging the revival of the juſtly-exploded doctrine of occult qualities *.

The ſentiments of the mechanical philoſophers may be found in the writings of Bacon, Boyle, and Newton.

In the preſent day, the chymical hypotheſis, as delineated by Dr. Crawford, Dr. Gardiner, and others, ſeems to give almoſt general ſatiſfaction on this curious ſubject; but it may be doubted whether this ſyſtem has yet been verified. How far the following obſervations will coaleſce with a chymical ſolution, is ſubmitted, with all deference, to thoſe ingenious philoſophers.

### SECTION II.

1. Heat, as it reſpects a living animal, requires to be accurately diſtinguiſhed: firſt, into that ſtate of the body which produces a ſenſible effect

* That the blood, as a *living* fluid, is eſſentially warm, when contained in the veſſels of a living animal, is an opinion as old as Hippocrates. Thoſe who wiſh to ſee the ideas of Ariſtotle effect on the thermometer; fecondly, it is to be regarded as a *fenfation*, which the application of certain irritating fubftances will excite in the mind. Thefe two effects are neither neceffarily co-exiftent, nor neceffarily difunited. Menfurable heat may exift where there is a fenfation of intenfe cold, and *e contrario* *.

2. Confiderable divifion of the fkin by a fcalpel; the application of lapis infernalis; certain difeafes, and hot iron, will excite perceptions nearly fimilar: and it is a well-known fact, that, during the cold fit of an ague, the body of the patient often exceeds its natural temperature.

3. Learned men, who have attended to phenomena like thefe, fometimes produce them as inftances of the fallacy of the fenfes; and they think this deception is demonftrated by the application of the thermometer. But it may be properly inquired, wherein is the patient deceived? It is not in the *fenfation* that is excited; for if fuch a perception exifts in his mind, to

Ariftotle and others upon this fubject, may find them in Harvey's book de Generatione Animalium, Exercitat. 71. De Calido innato. See alfo De Haen's Ratio Medendi.

* Ant. de Haen Pars altera Rationis Medendi, Tom. I. Vindobonae. Edit. 1757.

affirm affirm its prefence cannot be a miftake. Should fuch a perfon infer that thefe fenfations are produced by the application of fire or ice, and that confequently his body can either elevate or deprefs the mercury in the thermometer, the conclufion may be unjuft, but the fenfation is certainly true. Properly fpeaking, the informations derived from the organs of fenfe, in a healthy ftate, are always true; but the inferences are often falfe. As this is true in fpeculation, fo it is juft in practice. When a mutation is produced in the nervous fyftem by difeafe, fimilar to that which is caufed by the application of fubftances fenfibly hot or cold, whatever be the language of our philofophical inftrument, the *fenfations* of the patient muft be ferioufly attended to in all our attempts to counteract the morbid condition of the fyftem.

4. The fenfation of heat may, therefore, be excited in a living body by difeafe, and by the application of certain irritating fubftances; and the fenfation of cold, by fuch morbid affections of the fyftem, as confiderably diminifh the fenfibility of the furface *.

5. Ought

* Infenfilitas—miferos aegros, dum hypocauftulis pedibus applicatis moleftum frigus lenire vellent, ad offa ufque plantas pedum.

5. Ought not heat therefore, as it refpects a living animal, to be regarded as a fecondary quality, the *perception* of which does not *neceffarily* depend upon the tranfmiffion of any fluid from the fubftances applied, but is relative to the fpecies of impreffion made upon the organs of fenfe * ?

### SECTION III.

1. The chymical explanation of the mode in which animal heat is generated, feems to proceed upon the following principles :

A certain fluid, called elementary fire, is combined with atmofpheric air : when air, thus impregnated, is inhaled into the lungs, the phlogifton, in union with venous blood, decompofes it, and a double elective attraction takes place; the elementary fire is then conveyed into the blood veffels, and gradually evolved by the action of the vafa minima. The variations from this doctrine, propofed by Dr. Gardiner, chiefly relate to the mode of evolution †.

2. Two pedum combuffiffe abfque ullo doloris fenfu. —Van Swieten, Comment. in Herm. Boerhaave Aphor. Tom. II. fect. 576.

* Locke on Human Underftanding, chap. 8.

† This explanation is by no means fo novel as many have fuppofed; the general idea is fimilar to that publifhed feveral

 years

2. Two things are here aſſumed as data:—firſt, that there exiſts an element of fire: ſecondly, that the tranſmiſſion of blood through the lungs is abſolutely eſſential to the production of animal heat.

Has the exiſtence of fire, as *an element*, ever been demonſtrated?

To aſcend from effects to cauſes is certainly proper; and a ſucceſſion of certain phenomena, in a certain order, may enable us to form a law of nature; but if their cauſe be ſo ſubtile, that it cannot become the ſubject of phyſical inveſtigation, is it proper to affirm any thing concerning the *nature* of that cauſe?

Have we any better evidence of the exiſtence of an igneous fluid than of the exiſtence years ago by Mr. Freke, but now unfolded in a more elegant manner.

"All animals are in fact a fire engine; for as ſoon as the "lungs have received an inſpiration from the common air, "that fire which is ever found in all air will be inſtantly diſ-"perſed through the pulmonary veſſels into the blood; and "as that blood is ever nouriſhing and refreſhing ſome parts "with ſome of it, and imparting its fire through the nerves, "&c. the conſequences muſt follow, that the lungs hereby "becoming deprived of their uſual quantity, and deſirous of "that which every ſtone and log of wood deſires and receives,

"why

ſtence of that æther mentioned by Sir Iſaac Newton*?

If animal heat depends upon ſuch a proceſs as we have exhibited, the generation of heat in bodies, from friction, ought to ariſe from a ſimilar cauſe, or elſe there will be two different cauſes aſſigned for the ſame effect; but if rapid vibrations can liberate the igneous fluid, then it is not in chymical union with the ſubſtance from which it is evolved. Elementary fire, taken into the blood, and converted into ſenſible heat by the action of the blood veſſels, ought to produce its effects wherever the blood circulates, independently of any changes that may take place in the ſenſibility of the body: but that this is not true in fact will be preſently demonſtrated.

### SECTION IV.

1. In many caſes of partial paralyſis, where there was a loſs of ſenſibility in the part, but the motion remained, and the radial artery

"why may not the lungs become as active to reach and expand themſelves for more as often as they are robbed of it?" Freke on Fire, page 56.

* See Queries at the end of his Optics, and his Letter to the Hon. Robert Boyle, in Mr. Boyle's life.

contracted with vigour, there was a preternatural coldneſs in the limb, ſenſibly affecting the thermometer. So, on the contrary, where the pulſe has been totally imperceptible, not only in the wriſt, but in the arm and axilla, the heat has not been below the natural ſtandard *.

Now, if the evolution of fire from the blood were the only cauſe of animal heat, ought not this cauſe to operate wherever the blood circulates; and where vaſcular action is apparently loſt, ought not the effect to ceaſe likewiſe?

2. In many fevers, it is not unuſual for the animal heat to be at the natural ſtandard at the moment of death; it will often increaſe after this period to 101°, and the body will remain from 100° to 85° during ſeveral hours, although the ſurrounding atmoſphere does not elevate the thermometer to 60° †. It is alſo a well-eſtabliſhed fact, that many who die of malignant fevers, after a very ſhort ſickneſs, are actually and ſenſibly cold for many hours before

* Memoires de l'Acad. Royale des Sciences pour l'année 1743.—Anton. de Haen, Pars tertia Rationis Medendi, cap. 3.

† Ant. de Haen, Pars quarta Rationis Medendi, cap. 6. —Van Swieten, Comment. in Aph. Herm. Boerhaave. Tom. prim. ſect. 85.

death; death; but, foon after they have expired, the body will become as warm as a healthy, living body, and maintain that ftate during feveral hours.

3. Thofe who are fuffocated by mephitic vapours, fuch as the vapour of burning charcoal, fermenting wine, &c. retain their heat for many hours after all the functions of life have apparently ceafed. M. Portal obferves, that it is fometimes *above* the natural ftandard. The lungs of fuch people are always found in a ftate of perfect expiration; and there is an accumulation of blood on the right fide of the heart *.

4. In the preceding cafes it appears evident, that the heat did not owe its prefence to the procefs of refpiration; nor can it be attributed to putrefaction, fince that procefs had not fenfibly taken place. Befides, fuch an explanation would be attended with difficulties not at all inferior to the foregoing ones.

5. There are cafes recorded by anatomifts of the higheft reputation, where perfons have lived not only for days, but for years, with a pulmonary artery, incapable of tranfmitting blood

* Mem. de l'Acad. Royale des Sciences, 1775.

through through the lungs; ſo that the circulation has been carried on through the foramen ovale, or a perforation in the ſeptum cordis *.

6. It may be urged, that a ſmall quantity of blood might perhaps paſs by the pulmonary artery, or that the bronchial artery or vein might perform the requiſite proceſs.

Without attempting a direct refutation of ſuch ſuppoſitions, a moſt deciſive reply may be given on chymical principles.

The difference in colour between venous and arterious blood is ſaid to ariſe from the action of atmoſpheric air in the lungs, where the former is deprived at once of its phlogiſton and darker appearance, and is made to aſſume a bright ſcarlet colour †. The patients who laboured under the mal-conformation noticed above, had a remarkable livid appearance, not only over the whole ſurface of the ſkin, but even on the tunica conjunctiva. If the former theory be true, this would ſeem to indicate ei-

* Morgagni, Epiſt, 17, art. 12.—Dr. W. Hunter, Medical Obſervations and Inquiries, Vol. VI.—London Medical Journal for 1785, Part 4th, Art. Books. Cajetanus Taconus.

† It is not here meant to be aſſerted or denied that ſuch a principle as phlogiſton exiſts.

ther ther that no phlogifton was feparated from the blood, or that the quantity evolved was too fmall to produce any alteration in the colour of venous blood; yet, under fuch circumftances, the functions of animal life, and the procefs of nutrition, &c. were continued in one cafe for thirteen years, and in the other during fifteen.

In two of the cafes upon record, the lungs appeared to be almoft impermeable to atmofpheric air; nor could it be difcovered, on the moft accurate examination, that the girl of fifteen fuffered any expanfion or diminution of the cavity of the thorax during refpiration.

7. If, in certain circumftances, life and heat can be fupported for a confiderable time without the tranfmiffion of blood through the lungs, or expofure of that fluid to atmofpheric air, and confequent detachment of phlogifton, ought we to admit that thofe hypothefes have truly affigned the caufe of animal heat which affume a neceffity of refpiration and decompofition as data*?

* Some judicious obfervations on the different hypothefes are contained in a memoir written by M. de la Place, in the Mémoires de l'Académie Royale des Sciences for 1780.

8. It muſt be granted that inductive reaſoning is that ſpecies alone which ought to be admitted in the " interpretation of nature ;" and it is very probable that the great Lord Bacon, who reformed philoſophy, was capable of following his own rules : — has the explanation he has given of the "*form of heat*" ever been diſproved by experiment and induction ?

Intellectus humanus ex proprietate ſua facile ſupponit majorem ordinem et aequalitatem in rebus, quam invenit : et cum multa ſint in natura monodica et plena imparitatis, tamen affingit parallela, et correſpondentia, et relativa, quae non ſunt. — Hinc elementum ignis — introductum eſt ad conſtituendam quaternionem cum reliquis tribus, quae ſubjiciuntur ſenſui.—

Novum Organum, Lib. i. aph. xlv.

*Air Street,*
April 20, 1786.

